# 

**Published:** 1858-03

**Authors:** 


					﻿PUBLISHED MONTHLY.
VOL. L]
[No. 3.
CHIc4Gq
MEDICAL JOURNAL
■CITED BT
1ST _ S. ZD.A.'VIS,	ID.,
ABD
'W. H. BVFORD, ZkdZ.JD.
W4RCH, 1838.
BARNET & CLARKE, BOOK AND JOB PRINTERS, “CHRONOTYPE” OFFICE,
188 LAKE STREET, CORNER OF WELLS.
P. O. Box 2293,]	[Chicago, Ill.
AT $2 PER ANNUM, IN ADVANCE.
				

